# Effect of Dietary Supplementation of Chestnut and Quebracho Tannin Supplementation on Neonatal Diarrhoea in Preweaning Calves

**DOI:** 10.3390/antiox13020237

**Published:** 2024-02-15

**Authors:** Matteo Dell’Anno, Sara Frazzini, Irene Ferri, Susanna Tuberti, Elisa Bonaldo, Benedetta Botti, Silvia Grossi, Carlo Angelo Sgoifo Rossi, Luciana Rossi

**Affiliations:** 1 Department of Veterinary Medicine and Animal Sciences—DIVAS, Università degli Studi di Milano, 26900 Lodi, Italy; matteo.dellanno@unimi.it (M.D.); sara.frazzini@unimi.it (S.F.); irene.ferri@unimi.it (I.F.); susanna.tuberti@studenti.unimi.it (S.T.); elisa.bonaldo@studenti.unimi.it (E.B.); silvia.grossi@unimi.it (S.G.); carlo.sgoifo@unimi.it (C.A.S.R.); 2 Freelance Veterinarian, Via Alessandrini, 4, Bogolese di Sorbolo, 43058 Parma, Italy; benedetta.botti@gmail.com

**Keywords:** natural extracts, polyphenols, phytochemicals, antioxidants, antimicrobial, alternative to antibiotics, *Cryptosporidium parvum*, *Castanea sativa*, *Schinopsis* spp.

## Abstract

Neonatal calf diarrhoea (NCD) poses a significant health challenge in cattle herds, resulting in considerable economic losses and antimicrobial use. In response to the escalating threat of antimicrobial resistance, viable alternatives are imperative, aligning with European policies. This study evaluated the in-milk supplementation of the chestnut and quebracho tannin extract in preweaning calves on performance, diarrhoea occurrence, *Cryptosporidium* spp. shedding, protein digestibility, and intestinal health. Twenty newborn calves were divided, after colostrum administration, into two experimental groups for 30 days as follows: the control (CTRL) was fed with whole milk and solid feed, and tannins (TAN) were fed whole milk supplemented with 6/g day of tannin extract and solid feed. Faecal samples were collected on days 0, 3, 7, 14, and 30 for the evaluation of *Cryptosporidium* oocyst shedding and protein digestibility. Faecal consistency was evaluated during the sampling using the faecal score scale (0–3 scale, considering diarrhoea > 1). The results showed a significant reduction in diarrhoea frequency in the TAN compared to the CTRL group (*p* < 0.05) over 30 days of the trial. The prevalence of *Cryptosporidium* spp. was generally low (12%), considering all analysed samples. Protein digestibility revealed comparable values for the TAN and CTRL groups, suggesting that tannins did not negatively affect milk protein availability. In conclusion, the in-milk supplementation of 6/g day of the chestnut and quebracho tannin extract could be considered a valuable functional feed additive to decrease NCD occurrence, thus supporting animal health and decreasing antibiotic use in livestock.

## 1. Introduction

Neonatal calf diarrhoea has an important impact on economic losses in dairy farms. It is estimated that diarrhoea is one of the primary causes of mortality in calves (53–57%) [1]. Moreover, the high morbidity, mortality, and antibiotic treatments associated with NCD occurrence significantly affect animal health and future dairy performance during the first lactation [2]. This disease is defined as multifactorial, where the interaction among microorganisms, the host, and the environment plays a pivotal role in its determinism. The main pathogens involved in NCD are *Rotavirus*, *Coronavirus*, *Escherichia coli* K99, *Salmonella* spp., *Clostridium perfrigens* and *Cryptosporidium parvum* [3,4]. In particular, *C. parvum* is a protozoan etiological agent of enteric infections and diarrhoea in various mammalian species, with studies estimating its prevalence ranging from 50 to 100% in dairy cattle worldwide [5,6], indicating a significant and widespread occurrence.

*C. parvum* is characterized by high resistance to different environmental conditions and high contagiousness [7]. The oocysts of *C. parvum*, after ingestion, release sporozoites, which colonises intestinal villi, establishing the clinical symptoms [8]. The infection often occurs after 3 days of life, reaching a peak of incidence 4–18 days after birth, depending on the nutritional and immune status of the calf [9]. The parasite causes diarrhoea with possible complications due to metabolic acidosis and an electrolyte unbalance, which can lead to severe dehydration and death in some cases [10]. Even if not always lethal, the *C. parvum* infection alters the gut barrier integrity and the absorption ability causing diarrhoea and facilitating invasion by other pathogens [11]. In the context of NCD, which significantly impacts economic losses in dairy farms, *C. parvum* is a noteworthy contributor.

Animals affected by NCD require treatment with antimicrobial drugs. Nevertheless, the growing issue of antimicrobial resistance in the livestock sector underlines the need for substitutes and to reduce the use of veterinary drugs in food-producing animals. Recently, this phenomenon prompted the European Union to introduce more limitations on veterinary drug use [12]. Nutrition plays a pivotal role in the health and welfare of humans and animals, and it is no longer intended only to satisfy nutritional requirements [13]. The supplementation of functional feed additives such as probiotics, prebiotics, phytochemicals, organic acids, and essential oils can sustain the health status and reduce the risk of pathologies in livestock [14]. Tannins are a polyphenolic class of secondary metabolites with well-known antioxidant, anti-inflammatory, and antimicrobial activities [15]. They can be classified as hydrolysable (pyrogallol) or condensed tannins (proanthocyanidins) [16]. Hydrolysable tannins can be hydrolysed in monomers by chemical or enzymatic treatments. These are composed of phenolic acids and polyols (commonly glucose) grouped into gallotannins and ellagitannins [17]. Condensed tannins (1–30 kDa) are more abundant in plants compared to hydrolysable ones (500–3000 Da) [18].

The efficacy of tannins as antioxidants stems from the presence of phenolic hydroxyl groups, which facilitate electron donation to quench reactive oxygen species and mitigate oxidative damage [19]. Extensive research has established the ability of tannins to scavenge free radicals, providing cellular protection against oxidative stress [20,21,22]. Moreover, their anti-inflammatory and anti-cancer properties make them subjects of interest in therapeutic applications [23]. The diverse dietary sources of tannins, such as tea, fruits, and nuts, emphasize their potential as natural antioxidants for human and animal health [24,25]. Tannins can inhibit the growth of several microorganisms, showing both a bactericidal and bacteriostatic effect depending on their concentration, class of tannins, and structural properties. An important antimicrobial property of tannins is their capacity to inhibit cell wall synthesis by directly binding to peptidoglycan, destroying the integrity of the bacterial wall and increasing the susceptibility to osmotic lysis [26]. In addition, tannins can affect the membrane potential, increasing the permeability of bacterial cell membranes and leading to cell death. Some classes of tannins can interact with the lipopolysaccharides contained in the membrane of Gram-negative bacteria. Proanthocyanidins have been shown to bind with lipopolysaccharides, impairing membrane integrity [27].

The use of moderate concentrations of tannins in ruminant nutrition (0.2–0.6% of dry matter in steers and 10 g/day for calves) was shown to improve animal performance and decrease gastrointestinal parasitism and nitrogen pollution [28,29,30]. However, the effect of tannin supplementation in the liquid feeds (whole milk and milk replacer) of calves was investigated by a limited number of studies with controversial findings. Krueger et al. [31] showed no effect of tannin supplementation on the growth performance and feed efficiency in calves. Demarco et al. [32] reported that the combination of tannins and probiotics increased the feed conversion without affecting growth performance and reduced the haptoglobin concentration after transportation in beef calves. The inclusion of 4 g/day of hydrolysable tannins in milk significantly raised the growth and antioxidant capacity of plasma and lowered diarrhoea occurrence and faecal shedding of pathogens in preweaning calves [33]. Soleiman et al. [34] highlighted that the supplementation of 4, 6, or 12 mg/L of tannic acid decreases the faecal score in Holstein calves.

However, tannins are reported to have the ability to bind dietary proteins, forming insoluble complexes, thus reducing their bioavailability for gut absorption and decreasing animal performance [35]. Therefore, it is clear that the negative impact of tannins on ruminants is not specific to the type of tannin but may depend on their inclusion rate [36]. Recent studies showed that chestnut and quebracho tannins could affect feed palatability, digestibility, and the utilization of dietary protein in swine [16,37]. The aim of the following study was to evaluate the effect of dietary supplementation of 6 g/day of the chestnut and quebracho tannin extract in milk on diarrhoea occurrence, *C. parvum* shedding, and protein digestibility in preweaning calves.

## 2. Materials and Methods

### 2.1. Animal Housing and Experimental Design

The experimental trial was approved by the Animal Welfare Organization of the University of Milan (OPBA_03_2021) and performed on a commercial dairy farm involved in the consortium of Parmigiano Reggiano (Emilia Romagna, Italy) in line with European regulations [38,39].

Within three hours of birth, twenty Holstein calves (7 males and 13 females; 44.25 ± 3.97 kg) were fed with 4 L of high-quality colostrum (checked with a refractometer ≥22 °Brix, ≥50 g/L of IgG) by bottle feeding with two portions. The day after, animals were allotted into two groups, balanced per weight: the control group (CTRL, *n* = 10) fed whole milk, and the tannins group (TAN, *n* = 10) fed whole milk supplemented with 6 g/day per calves of chestnut (*Castanea sativa*) and the quebracho (*Schinopsis* spp.) tannin extract (Silvafeed ByPro, Silvateam S.p.A., Cuneo, Italy; with a total phenol concentration of 0.70 g/g of tannic acid equivalent [40]; the feed additive was approved by the Reg. EC 1831/2003, identification number following Reg. EC 767/2009: IT000431CN) solubilized in a premixture of 50 mL of polypropylene glycol for 30 days. Dietary treatments were provided after colostrum administration, animals were fed whole milk twice per day, and ad libitum access to solid feed and water was guaranteed. In particular, calves were fed 10% of their body weight in milk for the entire trial [41]. Animals were housed in individual straw-bedded pens (0.90 × 1.80 m) with free access to water under homogeneous environmental conditions for 30 days.

### 2.2. Zootechnical Performance, Diarrhoea Frequency and Sample Collection

Body weight (BW) was individually recorded on days 0 (d0), 14 (d14), and 30 (d30). The average daily gain (ADG) was calculated by dividing the weight gain for the considered time period. Faecal consistency was scored on a daily basis for each animal using a four-point scale (0 = dry, 1 = normal, 2 = runny, 3 = watery), considering > 1 as diarrhoea [42,43]. The frequency of diarrhoea was evaluated as the percentage of observations of animals with clinical signs of diarrhoea divided by the total observation performed for the considered period (diarrhoea frequency = n° of faeces with score 2 and 3/total number of observations; moderate diarrhoea frequency = n° of faeces with score 2/total number of observations; severe diarrhoea frequency = n° of faeces with score 3/total number of observations).

Faecal samples were collected from the rectal ampulla on days 0 (d0), 3 (d3), 7 (d7), 14 (d14), and 30 (d30) for the evaluation of protein digestibility and *Cryptosporidium* spp. oocyst count via optical microscopy after floatation in 10 randomly selected observation fields at 400× magnification.

At d0 and d30, blood samples were collected from the jugular vein using vacuum tubes without anticoagulants for immunoenzymatic analyses.

### 2.3. Tannin Solubilization and Evaluation of Antioxidant Activity of Tannins Premixture

Every 7 days, a tannin-soluble premixture was prepared in order to facilitate the in-milk dispersion of the tannin extract during the individual milk-feeding procedure. Chestnut and quebracho tannin extract was diluted in a 20% propylene glycol solution in order to obtain a final concentration of 120 mg/mL, and 50 mL aliquots of premixture were prepared for daily administration in milk. Aliquot samples from the tannin premixture were collected at 0, 1, 2, 3, 5, 7, and 10 days from preparation for the evaluation of the antioxidant capacity stability over time.

The antioxidant capacity was assessed using the Trolox Equivalent Antioxidant Capacity assay according to Frazzini et al. [44]. Briefly, the reaction mixture with 2,2′-azino-bis (3-ethylbenzothiazoline-6-sulfonic acid) (ABTS) was generated by combining 5 mL of 7 mM ABTS with 88 µ of 140 mM of potassium persulfate (K_2_S_2_O_8_). After 16 h of incubation of the reaction mixture in the dark at room temperature, the working solution of ABTS^•+^ radical cation was obtained diluting ABTS^•+^ with deionized water until reaching 0.700 ± 0.02 of absorbance at 734 nm, room temperature, using a spectrophotometer (Jasco V-630 UV-Vis, Jasco Deutschland GmbH, Pfungstadt, Germany). Trolox (6-hydroxy-2,5,7,8-tetramethychroman-2-carboxylic acid) was used as a standard for the generation of a calibration curve from 2000 µM to 125 µM via serial dilution. The test was performed in triplicate by adding 10 µL of the diluted sample to 1 mL of the ABTS^•+^ working solution. Absorbances were measured after 6 min of incubation at room temperature, and the total antioxidant capacity was expressed as μmol Trolox Equivalent/g of tannin extract powder (μM TroloxEq/g).

### 2.4. Nutrient Composition of the Whole Milk

The milk used in this study for animal feeding was analysed in terms of the principal nutrients according to the Official Methods of Analysis [45]. In particular, the dry matter (DM), crude protein (CP), ether extract (EE), and ash content were determined. DM was obtained by drying whole milk in a forced air oven until reaching weight stability after two subsequent attempts at weighing (AOAC method 930.15). CP was determined by measuring the total nitrogen concentration in milk samples multiplied by 6.25 as the conversion factor according to the Kjeldahl method (AOAC method 2001.11). EE was determined using a butyrometer according to the Gerber method (AOAC method 2000.18). Ash content was obtained by incinerating dried milk samples in a muffle furnace at 550 °C (AOAC method 942.05). Each determination was performed in triplicate.

### 2.5. Faecal Parameters and Apparent Total Tract Digestibility of Dietary Protein

DM and nitrogen concentrations in faeces were measured according to the Official Methods of Analysis as previously described [45]. The apparent total tract digestibility of the dietary (ATTD) protein was evaluated using acid-insoluble ash (AIA) as a marker on milk and faecal samples. Pre-dried samples of milk and faeces were incinerated in a muffle furnace at 550 °C for 3 h (AOAC method 942.05). Ashes were subsequently diluted in HCl (3N) and boiled for 15 min. Residual ashes were filtered on paper filters (Whatman 41, Cytiva, Pall Corporation, New York, NY, USA) and washed with hot deionized water until a neutral pH was obtained using a litmus test. Filters were incinerated according to the previously described methodology for obtaining the weight of acid-insoluble ashes. The apparent total protein digestibility was calculated according to the following equation:

$$Apparent\;protein\;digestibility\;(\%)=100\times \left(1-\frac{acid\;insoluble\;ashes\;in\;feed}{acid\;insouble\;ashes\;in\;faeces}\times \frac{nitrogen\;content\;in\;faeces}{nitrogen\;content\;in\;feed}\right)$$

### 2.6. Enzyme-Linked Immunosorbent Assay of Glucagon-like Peptide 2 and Diamine Oxidase in Serum Samples

Blood samples collected on days 0 and 30 were centrifuged (3000 rpm, 15 min, RT) to obtain the serum. Glucagon-Like Peptide 2 (GLP2) and Diamine Oxidase (DAO) levels were quantified in duplicate using enzyme-linked immunosorbent assay (ELISA) kits specific for bovine species, following the manufacturer’s instructions (Bioassay Technology Laboratory, Shanghai, China). After the addition of 50 µL of the stop solution, absorbances were measured with a microplate reader (Bio-Rad 680 microplate reader, Bio-Rad Laboratories, Inc., Hercules, CA, USA) at 450 nm, and concentrations were calculated according to the respective standard curves using CurveExpert 1.4 software.

### 2.7. Statistical Analysis

Obtained data were statistically analysed through JMP Pro 15^®^ (SAS Inst. Inc., Cary, NC, USA) software. The antioxidant activity of the tannin premixture was evaluated with an analysis of variances (one-way ANOVA). The results of growth performance, faecal parameters, protein digestibility, and serum levels of GLP2 and DAO were analysed using a linear model, including the fixed effect of treatment (Trt), time (Time), and the interaction between treatment and time (Trt × Time), while each animal was included as a random factor. Pairwise comparisons were evaluated using Tukey’s Honestly Significant Difference test (Tukey’s HSD). Total ADG (0–30 days) was analysed using Student’s unpaired *t*-test. The frequencies of the faecal score were converted into a dichotomous variable (normal/pathological) considering diarrhoea for a registered faecal score > 1, moderate diarrhoea frequency for each faecal score = 2, and severe for a faecal score = 3. The observed frequencies were assessed using the chi-squared test, allowing us to evaluate whether differences between observed and expected frequencies were caused by treatments or only by casualty. The results are presented as means ± standard error. Means or frequencies were considered statistically different when *p* ≤ 0.05.

## 3. Results

### 3.1. Antioxidant Activity of Tannin Extract Premixture and Whole Milk Composition

**Figure 1:**
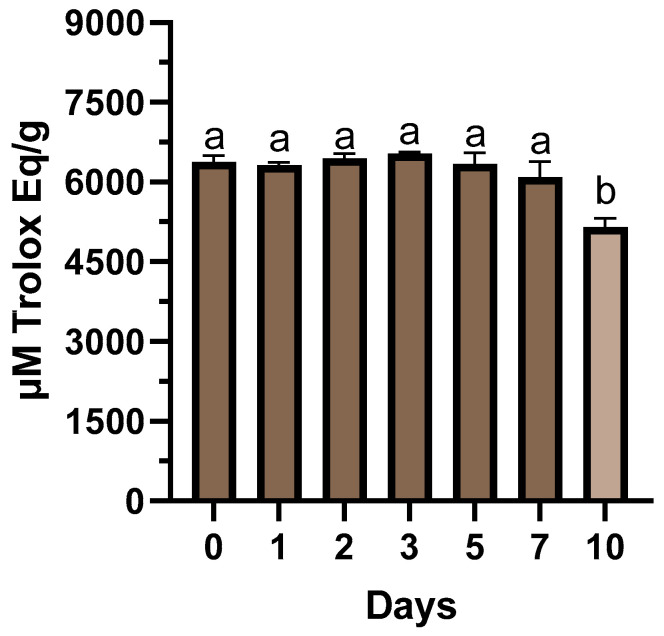
Antioxidant activity of tannins premixture (120 mg/mL in 20% propylene glycol solution) measured over 10 days of storage at room temperature. Data are presented as means ± standard error. ^a,b^ Different lowercase letters indicate statistically significant differences between days (*p* < 0.01).

The tannin premixture showed a stable antioxidant activity for 7 days. After 10 days from solubilization, the total antioxidant activity was significantly lowered to about 15.5% compared to 7 days after the preparation of the tannins premixture (Figure 1) (*p* < 0.01). The milk used for calf feeding showed a DM content of 12.98 ± 0.30%, CP 3.61 ± 0.04%, EE 4.28 ± 0.15% and 0.43 ± 0.14% of ashes.

### 3.2. Zootechnical Performance and Diarrhoea Occurrence

The calves’ growth showed comparable values for both groups over 30 days of the trial without registering significant differences in terms of growth performance (Table 1).

Diarrhoea occurrence showed a significant reduction in the TAN group compared to the CTRL during the 30 days of the trial (*p* < 0.05) (Table 2). A significantly lower frequency of moderate diarrhoea (faecal score = 2) was observed in the TAN compared to the CTRL group from 0 to 30 days (*p* < 0.05). The observed decrease was registered after 3 days of age, underlining −25% of moderate diarrhoea in the TAN-supplemented group (*p* < 0.05).

**Figure 2:**
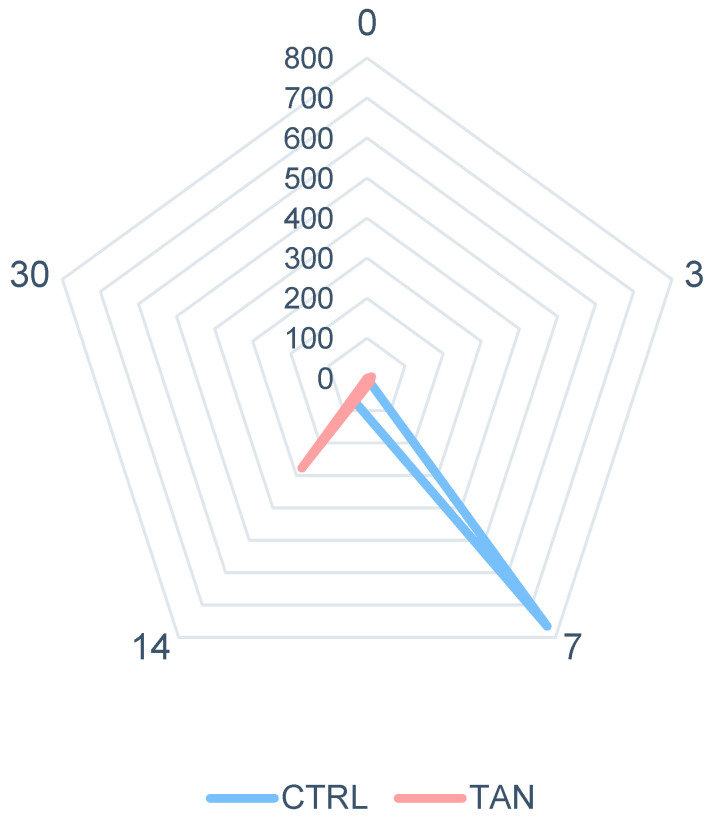
Spider plot of number of total faecal *Cryptosporidium* spp. oocysts measured in control (CTRL) and tannin (TAN) groups during the entire experimental trial of 30 days. Data are presented as the sum of total oocysts for each sampling timepoint (0, 3, 7, 14, and 30 days).

*Cryptosporidium* spp. showed that 50% of animals were positive for the presence of oocysts (4 from the CTRL and 6 from the TAN group); however, the prevalence of *Cryptosporidium* spp. was low (median value for both groups: 0) during the entire trial. The total number of measured oocysts was 828 for the CTRL and 302 for the TAN group, respectively. The presence of oocysts characterized the period from 3 to 14 days without registering any positivity on day 30 for both groups. The highest shedding was observed in the CTRL group after 7 days which registered 765 oocysts, while the TAN group registered a delayed peak at 14 days with 277 oocysts in faecal samples (Figure 2).

### 3.3. Faecal Parameters and Apparent Total Tract Digestibility of Dietary Protein

**Figure 3:**
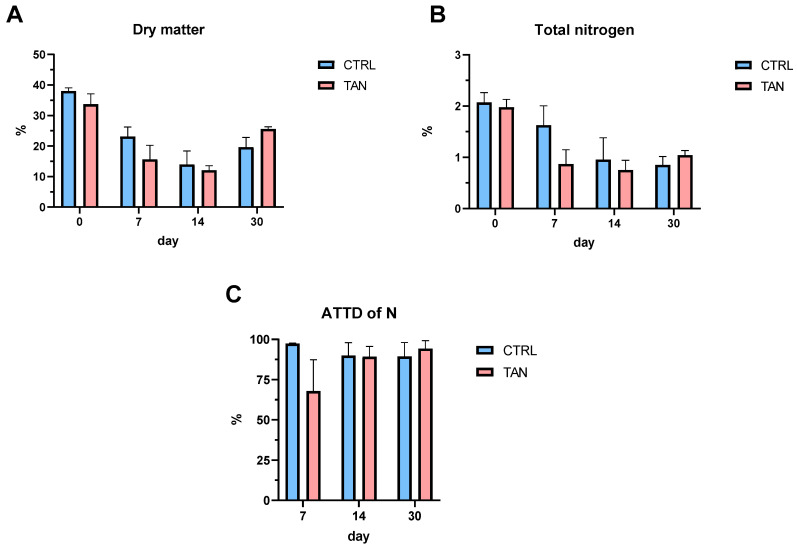
Faecal parameters and apparent total tract digestibility (ATTD) of nitrogen divided into control (CTRL) and tannin (TAN) groups at different timepoints of the experimental trial. (**A**) Dry matter of faecal samples at 0, 7, 14 and 30 days of trial; (**B**) Total nitrogen concentration of faecal samples at 0, 7, 14 and 30 days of trial on fresh matter basis; (**C**) Apparent total tract digestibility of nitrogen at 7, 14 and 30 days of trial. Data are presented as the means ± standard error.

Concentrations of dry matter and total nitrogen showed a comparable trend between the two groups during the experimental trial without underlining significant differences (Figure 3A,B). The ATTD of nitrogen displayed a high individual variability after one week of age without observing significant differences over the entire period (Figure 3C).

### 3.4. Serum Concentration of GLP2 and DAO

**Figure 4:**
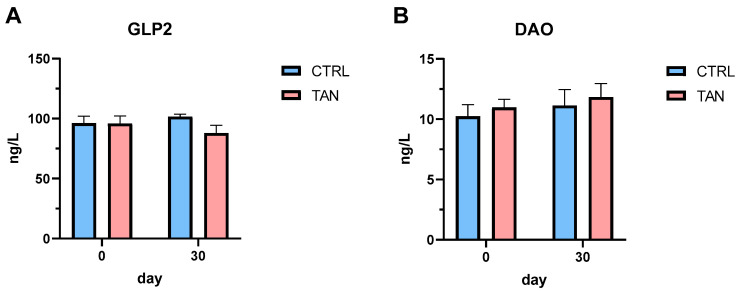
Serum concentration of blood metabolites titrated with enzyme-linked immunoassays in control (CTRL) and tannin (TAN) groups on days 0 and 30 of experimental trial. (**A**) Glucagon-like peptide 2 (GLP2) levels. (**B**) Diamine Oxidase (DAO) levels. Data are presented as means ± standard error.

Blood levels of GLP2 and DAO showed comparable values between the CTRL and TAN groups on days 0 and 30 of the experimental trial (Figure 4).

## 4. Discussion

Due to global concerns regarding antimicrobial resistance, there is a current demand for alternative treatments. Tannins extracted from different plants have been suggested for their potential to modulate rumen fermentation and reduce methane emissions in ruminants [46,47,48]. However, limited information is available on the impact of tannins on NCD and protein digestibility. The present study evaluated the effect of tannin extract supplementation in whole milk on calves’ performance, diarrhoea frequency, and *Cryptosporidium* spp. shedding, protein digestibility, and intestinal barrier status through indirect markers in serum. The tannin premixture showed high stability for 7 days after the preparation and registered a drop after 10 days. The registered antioxidant capacity was in line with the scavenging activity evaluated in our previous study on the tannin extract from chestnut and quebracho trees [49]. The liquid premixture optimized the procedure for the administration of 6 g/day of tannin extract within the individual milk-feeding procedure of the farm. This protocol ensured the preservation of antioxidant capacity for a week of storage, indicating this method is a practical and efficient approach for tannins supplementation in the diet of animals.

The nutritional content of the milk used in the present study exhibited nutrient levels similar to the Italian average for Holstein cow milk quality, especially in the context of Parmigiano Reggiano production [50,51,52]. The inclusion of tannins in milk in preweaning calves did not affect the growth curve over 30 days of the trial. A slight numerical difference was observed in the body weight of the TAN compared to the CTRL group after 30 days, even if not statistically significant. The lower average BW could be due to the presence of seven male calves in the CTRL group, which typically achieve more rapid growth compared to females. In line with our results, different studies have shown that tannin supplementation does not influence growth performance during the first month of life in calves [34,53,54]. Serri et al. [33] observed that the supplementation of 4/g day of the hydrolysable tannin extract in milk significantly improved ADG and final body weight after 76 days compared to the 2 g/day, 6 g/day, and control groups. Oliveira et al. [54] revealed that the tannin-rich pomegranate extract supplemented in feed significantly increased calves’ performance and feed intake from 30 to 70 days of age. These data suggest that regular tannin supplementation may have a positive impact on performance during the second month of life. Although it is challenging to directly compare natural extracts due to the significant variability in doses, batches, and the wide difference of commercial products on the market, the positive effects of tannins on calves’ performance could become more evident from the second month of life. 

The average age at which diarrhoea onset occurred was consistent with data reported in the existing literature [55,56]. The frequency of diarrhoea was significantly decreased in the TAN group compared to the CTRL during 30 days of the trial. In particular, moderate diarrhoea occurrence was reduced by 25% after 3 days from birth in the TAN compared to the CTRL group. Faecal *Cryptosporidium* spp. oocysts registered a low positivity (12% of total analysed samples) [57,58], suggesting that their presence was not the main cause driving diarrhoeic episodes. Only three animals (2 CTRL and 1 TAN) showed high parasitic shedding (>30 oocysts) according to the scale proposed by Delafosse et al. [59] on days 7 and 14 of the trial. Neonatal calves typically face an elevated susceptibility to gastrointestinal disorders, notably diarrhoea. Dietary strategies that reduce the probability of NCD are preferred, obviating the necessity for antimicrobial treatments in line with One Health principles. The intimate connection between oxidative stress and the immune system underscores the significance of maintaining a delicate equilibrium between oxidants and antioxidants within immune cells. This balance is crucial, as immune cells require the production of reactive oxygen species to complete their functions [60]. Plant extracts rich in polyphenols are extensively employed as feed additives, primarily due to their claimed beneficial effects associated with antioxidant and antimicrobial properties. Additionally, plant polyphenols have been reported for their ability to deactivate bacterial enterotoxin in vitro [61]. In this context, tannins are strong antioxidants that have been proposed as a valid substitute for substituting artificial antioxidants for different applications [62]. Numerous studies have suggested that oxidative stress characterizes pathological conditions such as NCD [63,64,65]; thus, dietary antioxidants could provide important support to restore oxidative balance. Consequently, raising the antioxidant capacity in newborn calves has the potential to promote immune system development, enhance health status, and consequently decrease calf mortality. The supplementation of tannins in animal nutrition is proposed as particularly advantageous for animal welfare and health and is primarily attributed to the enhancement of antioxidant status [33].

Tannins are also effective for their ability to inhibit bacterial growth and protease activity by damaging the cell wall and cytoplasm, causing rapid structural destruction. Previous studies have suggested that the antimicrobial effect of tannins may be related to their ability to impair microbial adhesions and inhibit hydrolytic enzymes such as proteases, carbohydrolases, and cell envelope transport proteins [62,66,67,68]. Consistent with our findings, several studies have reported enhanced faecal consistency following tannins supplementation, contributing to the reduction in the duration of diarrhoea, delaying its onset, and serving as a preventive measure in calves [29,33,53,69]. On the contrary, some studies did not register a preventive effect on diarrhoea [54,70]; however, a notable improvement in faecal consistency was recorded [54]. This difference could be linked to the broad range of tested doses of tannins and the different nature and composition of tannin extracts used in the literature. The extensive variation in dosages and diverse tannin extract profiles may influence the effectiveness of diarrhoea prevention, underscoring the need for a correct consideration of both the dosage and extract composition in feed applications.

The antiprotozoal ability of polyphenols against coccidia has been reported in previous studies [71,72,73], suggesting that tannins possess the ability to directly reduce the viability of the larval stage and disrupt egg hatching. Bhatta et al. [74] observed a reduction in protozoa when applying hydrolysable tannins from different plant sources. Benchaar et al. [75] reported that condensed tannins from quebracho can decrease protozoa abundance. In contrast, Vasta et al. [76] found that quebracho tannins were capable of increasing protozoa in rumen liquor. Discrepancies in the tannin concentrations, plant sources, protozoal species, and environmental factors considered in these studies may elucidate the conflicting results obtained, as these parameters significantly influence the antiprotozoal activity of polyphenols. Even if the recorded *Cryptosporidium* shedding did not reveal significant differences, a lower peak of oocysts in faecal samples was observed in the TAN compared to the CTRL group delaying its onset. The average number of days of *C. parvum* oocysts shedding was registered from 6 to 9 days of age in calves after experimental infection [57]. Zambriski et al. [58] observed a comparable faecal shedding pattern in calves regardless of the administered dose of *C. parvum* in experimentally challenged calves. Animals subjected to a lower oral dose delayed the shedding with a lower number of oocysts. We hypothesize that the antiparasitic properties of tannins could potentially limit the shedding of oocysts in calves, also causing a delay in faecal elimination. However, to comprehensively understand the impact of the combination of chestnut and quebracho tannins on calves affected by *Cryptosporidium* spp., further studies, including experimental challenges, will be necessary to provide a detailed examination of the interplay between these tannins and the parasitic infection.

Faecal DM, N concentrations, and the ATTD of dietary protein showed comparable levels in CTRL and TAN groups during the entire trial. Tannins are recognized for their pronounced affinity with proteins, giving rise to their astringent effect. By forming non-specific bonds with dietary proteins, tannins can create complexes resistant to gastrointestinal proteases. Unlike ruminants, tannins have traditionally been considered antinutritional factors in animal nutrition, leading to adverse effects on feed intake, nutrient digestibility, and growth performance [77]. Consequently, the feed industry aimed to minimize the inclusion of tannin-rich feed ingredients in the diets of pigs and poultry or to adopt measures for reducing their concentrations in the diet. However, recent studies have indicated that low concentrations of various tannin sources enhance the health status and performance of monogastric animals [16,78]. In the literature, only a few studies have assessed the effect of tannins on protein digestibility in calves. Soleiman and Kheiri [34] found that the supplementation of 4, 6, and 12 mg/L of tannic acid in milk did not influence the digestibility of protein and dietary lipids. The observed results align with registered growth performance, assuming similar milk digestibility. These findings present promising outcomes for supplementing the tannin extract into calf milk without compromising either milk palatability or the digestibility of the protein component.

No differences were observed for GLP2 and DAO serum concentrations after 30 days of tannin supplementation. GLP-2 is a 33-amino acid peptide primarily produced by intestinal L cells after food ingestion, derived from proglucagon (a hormonal peptide precursor). This hormone plays a critical role in the trophism of intestinal crypts, stimulating their proliferation and inhibiting apoptosis processes [79]. The GLP-2 increase in serum has been associated with improved morphology and intestinal functionality, positively impacting nutrient metabolism [80]. The observed values of GLP2 suggest that the supplementation of tannins did not negatively influence the production of this peptide by intestinal L cells; consequently, its role related to the trophism of intestinal crypts as a stimulator of proliferation and inhibitor of apoptosis remained unaffected after 30 days of the trial.

DAO is an antihistaminic enzyme produced by the intestinal epithelium of mammals. This enzyme is minimally present in serum under normal conditions. High serum levels of DAO may occur following damage to the intestinal mucosa; hence, the DAO concentration can be used as an indirect marker of intestinal integrity [81]. A previous study suggested that serum DAO levels may be modulated by feed additive supplementation in preweaning calves [82]. Serum DAO titres suggest that the animal welfare levels and health status were generally correct, in line with the absence of diarrhoea occurrence at the end of the trial.

The limitation of this study is that it relies on the short duration of the trial, which did not allow the evaluation of the long-term effects of tannins on growth and production performance. Furthermore, previous studies indicated the possible inhibitory activity of tannins on *Cryptosporidium* spp., but the low prevalence of this pathogen in our study did not allow us to emphasize this activity. However, our data provide interesting findings for practical applications. The use of 6 g/day of the investigated tannin extract could be considered a straightforward strategy to reduce the occurrence of diarrhoea, thereby enhancing both farm profitability and animal welfare. It is also important to underline the variability in feed additives available on the market, including differences in dosages and natural extract profiles. This variability makes it challenging to compare previous studies and establish a globally applicable protocol for alleviating NCD based on phytochemicals.

Considering the widespread issue of fake news in livestock farming, the need to share scientific findings through contemporary channels such as social media becomes fundamental. Recent studies conducted on Instagram as a tool for study engagement and information on the livestock system found that the viewing of the posts had a favourable impact on consumers’ opinions, even showing little change in participants’ attitudes [83,84]. These findings emphasize the importance of utilizing social media platforms for effective communication and outreach in animal science and veterinary education [85]. Furthermore, our study offers valuable suggestions and insights for creating social media content, contributing to the broader field of innovative communication strategies in animal and veterinary sciences.

## 5. Conclusions

The supplementation of 6 g/day of chestnut and quebracho tannin extract in milk did not influence the growth performance of calves over the course of 30 days. Tannins showed a positive impact on animal health by reducing diarrhoea frequency and improving faecal consistency. Throughout the trial, both the control and tannin groups did not show differences in the presence of faecal *Cryptosporidium* spp. oocysts. The tannin extract did not impair protein digestibility and milk palatability over the 30-day supplementation period. Further studies will be useful to comprehensively understand the specific effects of the chestnut and quebracho tannin extract on cryptosporidiosis. In conclusion, this study provides valuable insights, supporting the potential use of the combination of chestnut and quebracho tannins as functional feed additive to enhance health, reduce neonatal diarrhoea, and minimize antibiotic treatments in preweaning calves.

## Figures and Tables

**Table 1:** Growth performance of calves in control (CTRL) and tannin-supplemented group (TAN) during 30 days of trial.

	Group		*p*-Values		
	CTRL	TAN	Treatment	Time	Time × Treatment
BW (kg)			0.0417	<0.0001	0.9115
d0	45.30 ± 1.39	43.20 ± 1.39			
d14	45.80 ± 1.39	42.20 ± 1.39			
d30	60.80 ± 1.39	58.00 ± 1.39			
ADG (g/day)			0.7979	<0.0001	0.9423
d0–14	35.72 ± 79.90	35.71 ± 79.90			
d15–30	937.50 ± 79.90	893.75 ± 79.90			
d0–30	516.67 ± 63.25	493.33 ± 63.25	0.7971		

BW: body weight; ADG: average daily gain; CTRL: control group fed with whole milk; TAN: treatment group fed with whole milk supplemented with 6 g/day of tannin extract. Data are presented as means ± standard error.

**Table 2:** Diarrhoea frequency divided as presence (faecal score > 1), moderate (faecal score = 2), and severe (faecal score = 3) in calves of the control (CTRL) and tannin (TAN) groups registered during the 30 days of trial.

		CTRL	TAN	*p*-Value
Frequency of diarrhoea (%)	d0	10.00	0.00	0.1360
	d3	30.00 ^a^	5.00 ^b^	0.0191
	d7	36.00	25.00	0.3613
	d14	40.00	30.00	0.3291
	d30	20.00	15.00	0.6392
	d0–30	27.00 ^a^	15.00 ^b^	0.0150
Moderate diarrhoea (%)	d0	5.00	0.00	0.3049
	d3	30.00 ^a^	5.00 ^b^	0.0191
	d7	25.00	30.00	0.3613
	d14	35.00	25.00	0.3613
	d30	15.00	10.00	0.6056
	d0–30	22.00 ^a^	11.00 ^b^	0.0193
Severe diarrhoea (%)	d0	5.00	0.00	0.3049
	d3	0.00	0.00	-
	d7	10.00	10.00	1.0000
	d14	5.00	5.00	1.0000
	d30	5.00	5.00	1.0000
	d0–30	5.00	4.00	0.7268

Frequencies are presented as the percentage of observations of animals with clinical signs of diarrhoea for the considered period. ^a,b^ Different lowercase letters indicate statistically significant differences between groups (*p* < 0.05).

## Data Availability

Data are available within the article and from the corresponding author upon reasonable request.
